# Prediction of the mechanisms of action of Qutan Huoxue decoction in non-alcoholic steatohepatitis (NASH): a network pharmacology study and experimental validation

**DOI:** 10.1080/13880209.2023.2182892

**Published:** 2023-03-12

**Authors:** Xia Wu, Yurong Zhang, Ding Zheng, Yue Yin, Mengyun Peng, Jing Wang, Xiaoning Zhu

**Affiliations:** a Department of Integrated Traditional Chinese & Western Medicine, Southwest Medical University, Luzhou, China; b Hepatobiliary Department, The Affiliated Traditional Chinese Medicine Hospital of Southwest Medical University, Luzhou, China

**Keywords:** Kupffer cells, inflammatory response, traditional Chinese medicine, hepatocytes

## Abstract

**Context:**

Qutan Huoxue decoction (QTHX) is used to treat non-alcoholic steatohepatitis (NASH) with good efficacy in the clinic. However, the mechanism is not clear yet.

**Objective:**

This study investigates the mechanism of QTHX in the treatment of NASH.

**Materials and methods:**

Potential pathways of QTHX were predicted by network pharmacology. Fourty Sprague Dawley (SD) rats (half normal diet, half high-fat diet) were fed six to eight weeks, primary hepatocytes and Kupffer cells were extracted and co-cultured by the 0.4-micron trans well culture system. Then, the normal co-cultured cells were treated by normal serum, the NASH co-cultured cells were treated with various concentrations of QTHX-containing serum (0, 5, 7.5 or 10 μg/mL) for 24 h. The expression of targets were measured with Activity Fluorometric Assay, Western blot and PCR assay.

**Results:**

Network pharmacology indicated that liver-protective effect of QTHX was associated with its anti-inflammation response, oxidative stress, and lipid receptor signalling. 10 μg/mL QTHX significantly reduced the inflammation response and lipid levels in primary hepatocytes (ALT: 46.43 ± 2.76 U/L, AST: 13.96 ± 1.08 U/L, TG: 0.25 ± 0.01 mmol/L, TC: 0.14 ± 0.05 mmol/L), comparing with 0 μg/mL NASH group (ALT: 148 ± 9.22 U/L, AST: 53.02 ± 2.30 U/L, TG: 0.74 ± 0.07 mmol/L, TC: 0.91 ± 0.07 mmol/L) (*p* < 0.01). Meanwhile, QTHX increased expression of SOCS1 and decreased expression of TLR4, Myd88, NF-κB.

**Conclusions:**

The study suggested that QTHX treats NASH in rats by activating the SCOS1/NF-κB/TLR4 pathway, suggesting QTHX could be further developed as a potential liver-protecting agent.

## Introduction

Non-Alcoholic Fatty Liver Disease (NAFLD) is an obesity-related metabolic liver disorder with an estimated global prevalence of 24% (Younossi et al. 2018; Huang et al. 2021). Approximately 20% of patients with NAFLD progress to nonalcoholic steatohepatitis (NASH) (Li et al. 2019). NASH is defined by steatosis and inflammation with hepatocellular injury, inflammatory cells infiltration, hepatocyte ballooning degeneration with or without fibrosis and deteriorates to decompensated cirrhosis (DCC) and hepatocellular carcinoma (HCC) and may ultimately require liver transplantation (LT) (Mazhar 2019). The development of NASH is closely related to diabetes, obesity, hypertension, hyperlipidaemia, and metabolic syndrome (Sumida and Yoneda 2018). These metabolic-related diseases are also considered the main risk factors for NASH (Younossi et al. 2016; Oseini and Sanyal 2017). Therefore, it is important to find therapeutic ways to prevent and treat NASH (Pfister et al. 2021). Traditional Chinese medicine (TCM) is an important alternative medicine for many diseases and it has been developed over thousands of years with a unique system of theories, diagnostics, and therapies. The Qutan Huoxue decoction (QTHX) is mainly composed of *Pinellia ternata* (Thunb.) Breit (Araceae) (stem), *Citrus sinensis* (L.) Osbeck (Rutaceae) (peel), *Salvia miltiorrhiza* Bunge (Rutaceae) (peel), *Coix lacryma-jobi* L.var.*mayuen* (Roman.) Stapf (Poaceae) (seed), *Bupleurum scorzonerifolium* Willd (Apiaceae) (root), *Alisma plantago-aquatica* Linn. (Alismataceae) (stem and root), *Cassia tora* Linn. (Leguminosae) (seed), *Poria cocos* (Schw.) Wolf, (Polyporaceae) (Sclerotium), and *Scutellaria baicalensis* Georgi (Lamiaceae) (root). Our previous study has randomized 66 NASH patients into two groups for three months of therapy: QTHX (30 g/day) *n* = 33, and polyene phosphatidylcholine (784 mg/day) *n* = 33, the result suggested that QTHX appears to ameliorate the metabolic asset of mild NASH patients (Zheng et al. 2019). Previous animal studies showed that QTHX can significantly improve insulin resistance, hepatocyte injury, and liver fibrosis in NAFLD rats induced by a high-fat diet al.so, studies demonstrated the effect of the QTHX on expression of PPAR-α/CTP1, p38MAPK, AQP9, and SOCS1/TLR4/NF-κB pathway (Zheng et al. 2019; Zhang Y. et al. 2020), and others. Our study identified and verified the signalling pathway of QTHX treatment by merging pathological characteristics of NASH. The traditional Chinese medicine formula contains multiple herbal ingredients and these bioactive components target multiple genes and proteins. The latest research showed that suppressor of cytokine signalling-1 (SOCS1) deficiency increases macrophage infiltration (Ilangumaran et al. 2017). Thus, we speculate that QTHX may influence the disease progression of NASH by regulating the function of macrophages. In this study, a comprehensive method was used to illustrate the molecular mechanisms of QTHX. The primary hepatocytes and primary Kupffer cells were isolated and co-cultured from NASH model rats to establish an *in vitro* model of NASH, and systematically explore the mechanism of QTHX to treat NASH.

## Material and methods

### QTHX preparation

QTHX was prepared by The Affiliated TCM Hospital of Southwest Medical University. The quality of the herbs and herbal extracts was consistent with the standards of Chinese Pharmacopoeia. The medicinal materials were identified by the Professor Jing Wang and Dr. Xiaoning Zhu. Voucher specimens were prepared for identification and deposited in laboratory of Integrated Traditional Chinese and Western Medicine of The Affiliated TCM Hospital of Southwest Medical University. Medicines were prepared by boiling water extraction, decompression, concentration, and distillation filtration. The quality of QTHX was confirmed by ultra-high-performance liquid chromatography-tandem mass spectrometry (UPLC-MS/MS).

### Analysis of QTHX by network Pharmacology

All compounds of the Chinese medicinal herbs in QTHX were collected from the Traditional Chinese Medicine Systems Pharmacology Database and Analysis Platform (TCMSP) database (https://old.tcmsp-e.com/). The TCMSP database provides the pharmacokinetic properties of the QTHX chemical compound. Our study meets the requirements of oral bioavailability (OB) ≥ 30% and drug-likeness (DL) index ≥ 0.18. The NASH-associated genes targets were retrieved from the GeneCards, OMIM, PharmGkb, Therapeutic target, and DrugBank database and the proteins were retrieved from UniProt Knowledgebase (UniProtKB). The gene and drug target networks were constructed using Cytoscape. Based on the screened potential therapeutic targets, pathway and functional enrichment analysis of QTHX against NASH was analysed by GO and KEGG using R 3.6.1 BiocManager, Compound-target (C-T), compound-target-pathway (C-T-P) networks were constructed using the Cytoscape 3.6.0 software.

## Experimental verification

### Animals

Forty SPF (specific-pathogen-free) male Sprague Dawley (SD) rats (180 ± 20 g of body weight) were provided by the Experimental Animal Center of Southwest Medical University (SYXK (Sichuan) 2020-065). All rats were kept at 20 ± 2***°C*** with a 12 h light/dark cycle under specific pathogen-free conditions. Rats were randomly divided into the NASH and the control group. The NASH group rats were fed a high-fat diet (88% basic diet + 10% lard + 2% cholesterol, provided by Chengdu Dashuo Experimental Animal Company), and the control group was fed an ordinary diet (provided by Experimental Animal Center of Southwest Medical University). After six to eight weeks, two rats were randomly sacrificed from each group, for scoring the pathological changes in liver tissue according to the NASH scoring system (Table 1). All animal experiments were carried out by the guidelines of the Guide for the Care and Use of Laboratory Animals of the Institutional Animal Care and Use Committee (IACUC) set by The Affiliated Traditional Chinese Medicine Hospital of Southwest Medical University. The protocols were approved by the Animal Care and Use Committee of the Southwest Medical University (Luzhou, China) (protocol number: 20210218-006). Pentobarbital sodium was used as an anaesthetic to minimize pain during all procedures.

**Table 1.** NASH score.

Score	Fatty change (0–3)	Inflammation in the lobules (0–3)	Ballooning change (0–2)
0	<5%	None	None
1	5%–33%	<2	rare
2	34%–66%	2–4	more common
3	>66%	>4	–

*Note:* Criteria: NASH (fatty change + inflammation in the lobules + ballooning change) ≥4 points.

## Results

### Animal preparation

The methods of animal preparation and collagenase *in situ* liver perfusion were described by Peng et al. (2017).

### Isolation of primary hepatocytes and KCs

The liver was collected and carefully transferred into a 10 cm culture dish. It was minced into small pieces by scissors, after digestion, the cell suspension was filtered through a 70 μm tissue filter into a 50 mL centrifuge tube, and centrifuged at 50 *g* for 3 min to obtain the supernatant and pellets. The pellets were resuspended with a complete medium (10% foetal bovine serum, 1% penicillin solution, 500 U/L insulin, 10^-7^ M hydrocortisone, DMEM high glucose medium), and 50% Percoll was added (Solarbio, P8370-100 mL), centrifuged at 100 *g* for 5 min, discarded the supernatant. The cells were suspended in the complete medium and seeded into a cell culture dish, incubated at 37 °C in an atmosphere of 5% CO_2_-95% air. The medium was changed for the first time after 6 h. The obtained supernatant was centrifuged at 100 *g* for 5 min to obtain pellets, the supernatant was discarded, the pellet was resuspended, 50% Percoll was added slowly to the first layer, 25% Percoll dispersion to the second layer, and cell suspension to the third layer, centrifuged at 300 *g* for 15 min. The liquid was divided into four layers, the KCs located between 50% Percoll dispersion and 25% dispersion. The obtained KCs were resuspended in the DMEM media. The primary KCs were washed 3 times and the media was replaced after 2 h. Primary hepatocytes were identified by the anti-Cytokeratin 18 antibody (Abcam, ab133263) and microscopic morphological observation. KCs were identified by the CD68 Antibody (Santa Cruz Biotechnology, sc-17832) and ink swallowing experiment. Primary hepatocytes were co-cultured with primary KCs in a 0.4-micron trans well chamber. The upper chamber was inoculated with primary hepatocytes, and the lower chamber was inoculated with KCs. After 24 h, the cells were collected and culture fluid. In addition, the ratio of primary hepatocytes: primary Kupffer cells = 6:1 was used for co-cultivation.

### Cytotoxicity assay

QTHX-containing serum (QTHX-CS) was prepared according to the previously described method (Liu et al. 2017). Co-cultured cells were seeded into 96-well plates and treated with various concentrations of QTHX-CS (100, 80, 60, 40, 20, 10, 7.5, 5, 0 μg/mL) from the second day for 48 h. Cytotoxicity assay was performed using the Cell Counting Kit-8 (CCK8) (Dojindo, CK04) following the manufacturer’s instruction.

### Oil red O staining

The lipid deposition of primary hepatocytes was identified by oil red O staining (Solarbio, G1261-2): the culture medium was discarded and the cells were washed with PBS 1 ∼ 2 times, and they were fixed with 10% neutral formaldehyde for 30 min, stained with oil O red for 10 min. Next, 60% isopropanol was used for decolorization, followed by haematoxylin staining for 2–5 min, and the cells were viewed under a microscope. The isopropanol decolorization was used to quantitatively analyse the oil red O staining with a microplate reader.

### Liver function analysis

The contents of aspartate aminotransferase (AST), alanine aminotransferase (ALT), total cholesterol (TC) and triacylglycerol (TG) in primary hepatocytes were determined by using Activity Fluorometric Assay kit (Nanjing Jian Cheng Institute of Bioengineering, C 009-2, C 010-2) following the manufacturer’s instruction.

### Immunofluorescence staining

Cells were harvested and cytospined onto slides, washed twice with PBS, fixed with 10% neutral formaldehyde for 20 min, permeabilized with 0.2% Triton X-100 for 10 min, and blocked with 10% foetal bovine serum for 30 min. Then, the cells were incubated overnight with a rabbit anti-SOCS1 monoclonal antibody (Abcam, ab280886, 1:200 dilution) at 4 °C. Goat Anti-Rabbit IgG H&L (Alexa Fluor® 488) (Abcam, ab150077) was incubated in the dark after 1 h, DAPI (Solarbio, C0060-1) stained for 10 min, washed with PBS and observed under a fluorescence microscope.

### Real-time PCR

Total RNA from cultured cells was isolated using a Chloroform-free RNA extraction kit (BioTeke, RP55011) following the manufacturer’s instruction. Purified RNA was reversely transcribed into cDNA using the QuantiTect Reverse Transcription Kit (Qiagen, 208054) according to the manufacturer’s instructions. Quantitative real-time PCR was performed according to the kit instructions, β-actin was used as an internal reference, and the comparative cycle threshold method (CT) was used to detect the relative target gene mRNA expression. The primer sequences are shown in Table 2.

**Table 2.** qPCR primer sequence.

TLR4	F	TCCCTGCATAGAGGTAGTTCC
	R	TCAAGGGGTTGAAGCTCAGA
NF-kB	F	CCACTCTGGCGCAGAAGTTA
	R	CCCCCAGAGACCTCATAGTTG
Myd88	F	TCGACGCCTTCATCTGCTAC
	R	CCATGCGACGACACCTTTTC
SOCS1	F	CACGCACTTCCGCACATTCC
	R	TCCAGCAGCTCGAAGAGGGA
β-actin	F	CCCATCTATGAGGGTTACGC
	R	TTTAATGTCACGCACGATTTC

### Western immunoblotting

Cells were lysed on ice for 30 min. The lysate was separated by SDS-PAGE and transferred to the PVDF membrane. Cells were incubated with antibodies (TLR4, myd88, NF-κB, SOCS1, GAPDH, 1:1000) at 4 °C overnight. Next, they were incubated with an anti-rabbit or an anti-mouse antibody (1:5000). Chemiluminescence system was enhanced to detect protein bands, and the results were analysed by ImageJ 1.51 software.

### Statistical analysis

All data were analysed by GraphPad Prism 8.0. Values in the text are presented as means ± standard deviations (x ± s). Comparison among the three groups was performed by analysis of variance and pairwise comparison by the Shapiro–Wilk test. *p* < 0.05 indicates that the difference is statistically significant.

## Results

### Identification of the active compounds in QTHX

147 compounds and 1401 related targets were retrieved from the TCMSP database, as shown in Table 3, 414 known therapeutic targets for NASH were collected from the Genecards, OMIM, PharmGkb, TTD, and DrugBank databases. NASH treatment targets were mapped to ten drug targets in QTHX using the Venn R package (version: V3.6.1) to construct a Venn diagram, showing 38 common therapeutic targets of QTHX therapy NASH.

**Table 3.** The candidate active compounds of QTHX.

Chinese name	English name	Proportion (g)	Retrieve active ingredient	*Related targets*
Ban Xia	Rhizoma Pinelliae	10	13	172
Chen Pi	Pericarpium Citri Reticulatae	10	5	95
Dan Shen	Radix Salviae Miltiorrhizae	15	15	95
Yu Jin	Radix Curcumae	15	15	76
Yi Yiren	Coix Seed	15	9	48
Chai Hu	Radix Bupleuri	10	15	173
Ze Xie	Oriental Waterplantain Rhizome	15	10	24
Jue Mingzi	Cassia Seed	10	14	181
Ful In	Poria	10	15	30
Huang Qin	Radix Scutellariae	10	36	507

### GO and KEGG analyses of target genes

**Figure 1.**
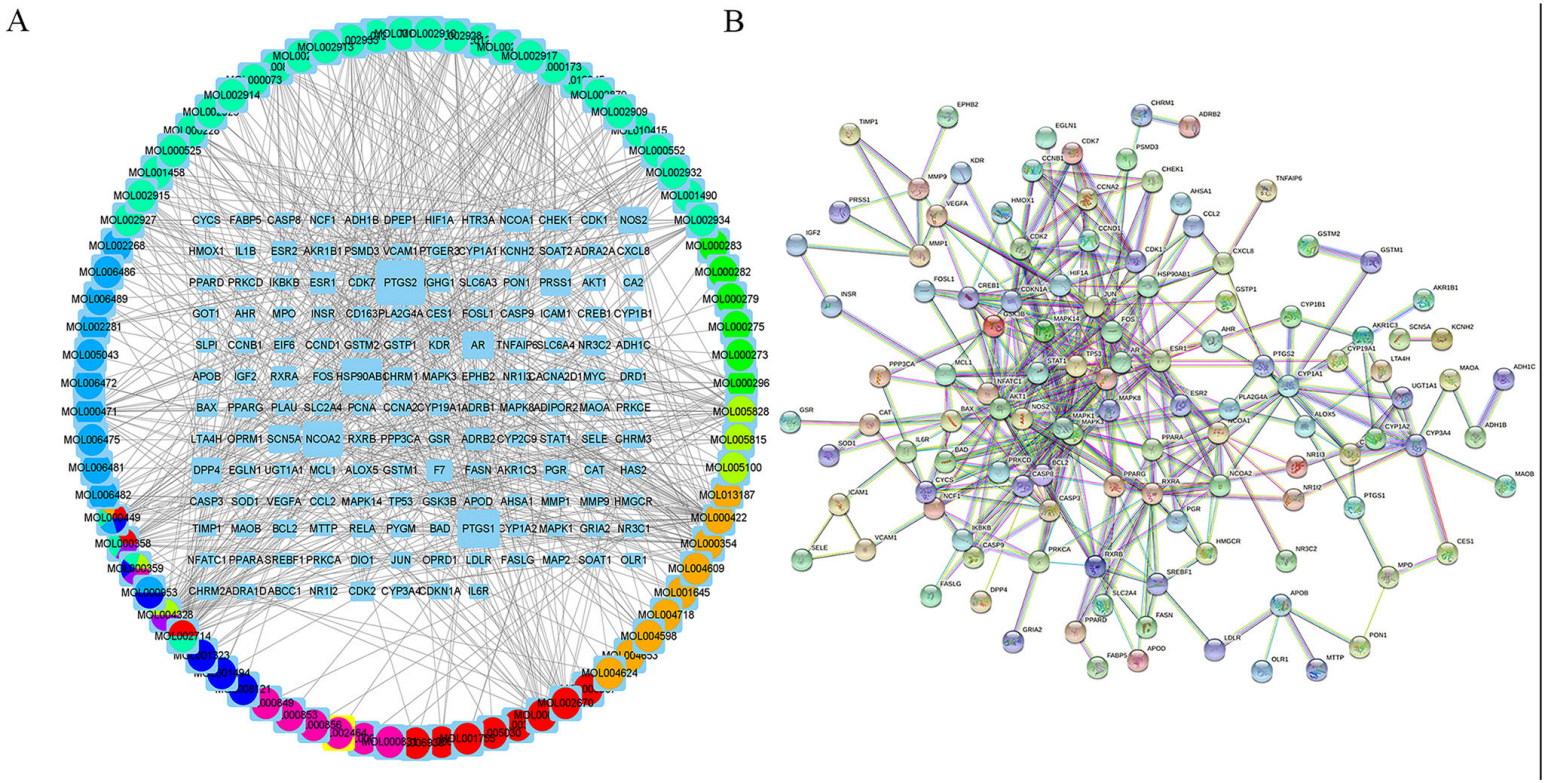
Disease-drug-ingredient-target network and PPI.

**Figure 2.**
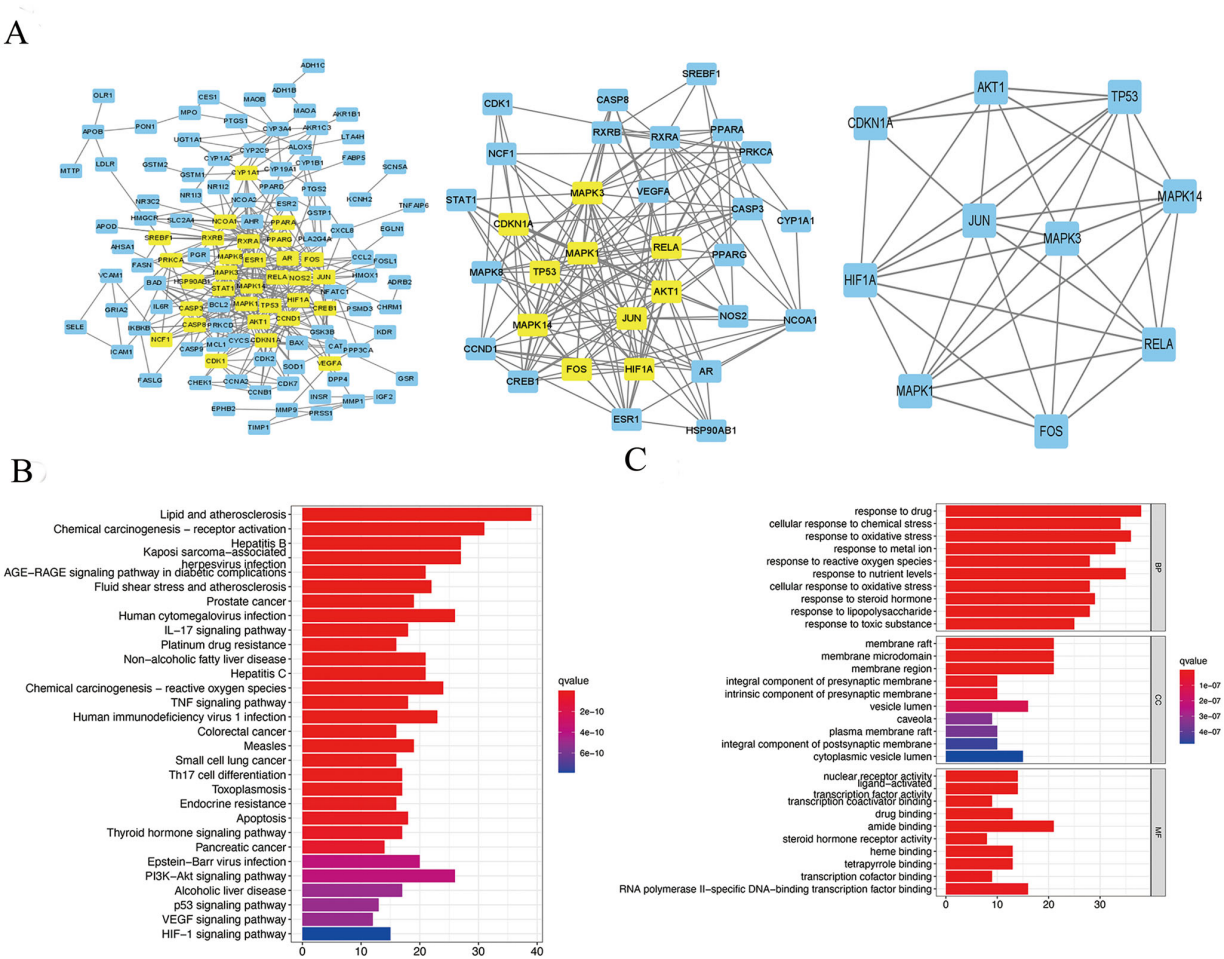
PPI core network and enrichment analysis. (A) PPI core network. (B) KEGG enrichment analysis. (C) GO enrichment analysis.

The disease-drug-ingredient-target interaction network was obtained from drug networks and it was constructed by Perl script and Cytoscape software (Figure 1(A)). The protein-protein interaction (PPI) network was illustrated based on the QTHX targets on NASH with the STRING database (Figure 1(B)) and the PPI core network (Figure 2(A)), was settled at 10 hub nodes, and 35 edges, which includes MAPK14, AKT1, FOX, HIF1A, RELA, JUN, TP53, MAPK3, CDKN1A, MAPK1. Then, metascape was used for GO and KEGG analyses of the 10 genes to obtain enriched ontology clusters (Figure 2(B,C)), the functional enrichment mainly included oxidative stress, membrane raft, membrane microdomain, membrane region, nuclear receptor activity, etc. The signalling pathway enrichment mainly included lipid and atherosclerosis, chemical carcinogenesis-receptor activation, human cytomegalovirus infection, etc. Therefore, it is speculated that the molecular mechanism of QTHX in the treatment of NASH may be related to inflammation and lipid deposition by targeting RELA, so the signalling pathway TLR4/NF-κB related to RELA was used to explore the specific mechanism of QTHX in the treatment of NASH.

### Isolation and co-culture of NASH primary hepatocytes and KCs

**Figure 3.**
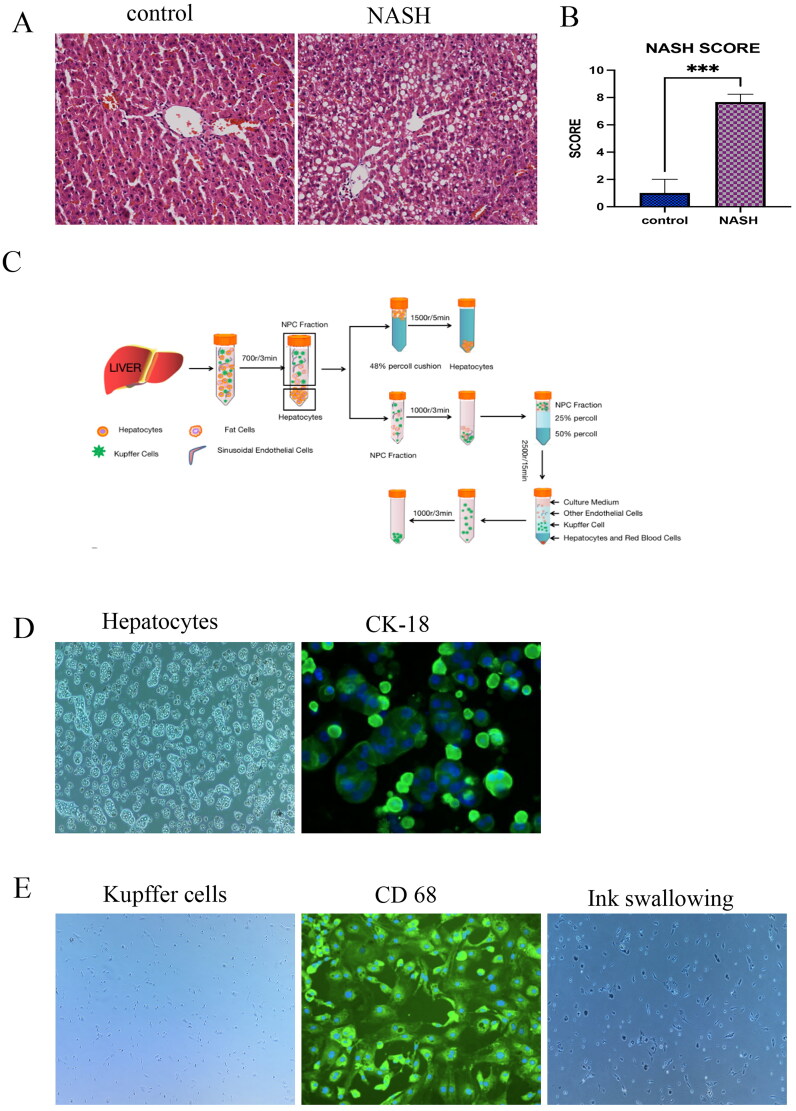
Isolation and co-culture of NASH primary hepatocytes and KCs. (A) the control group and NASH group HE staining of liver sections. (B) NASH score. (C) cell extraction process. (D–E) Under microscope, CD68 immunofluorescence images, ink swallowing experiment of rat primary KCs and under microscope, CK-18 immunofluorescence images of primary hepatocytes. ***p < 0.001.

To explore the specific mechanism of QTHX in the treatment of NASH, the primary Kupffer cells and primary hepatocytes were extracted and co-cultured from the NASH rats that have been fed a high-fat diet. The HE staining showed that the cells in the control group were intact, the hepatocytes were neatly arranged, and no fatty vacuoles were seen in the cells. However, in the NASH group, the hepatocytes were incomplete and not neatly arranged. The different sizes of cells were swollen and large vacuoles of triglyceride fat accumulated were found in the cells (Figure 3(A)), after NASH scoring, fatty degeneration score + intralobular inflammation score + ballooning score ≥4 points (Figure 3(B)). In this study, collagenase *in situ* perfusion was used to separate primary hepatocytes and primary KCs of rats (Figure 3(C)). This method could obtain primary hepatocytes about 1 ∼ 2 × 10^7–8^ cells/mL and primary KCs about 1 ∼ 2 × 10^5–6^ cells/mL. Observing the cells under an inverted optical microscope, the shape of primary hepatocytes was like irregular paving stones, KCs were seen to be round, or irregular in shape. The monoclonal antibody CK-18, identified that the primary hepatocytes showed positive expression, fluorescence evenly distributed in the cytoplasm, and no expression of other non-hepatic parenchymal cells was reported. In the ink swallowing experiment, KCs swallowed the ink and then turned black and the monoclonal antibody CD68 identified a positive expression (Figure 3(D,E)).

### QTHX regulates the TLR4/NF-κB pathway in vitro

The QTHX regulation of the TLR4/NF-κB pathway was investigated *in vitro*. QTHX-CS was added to a co-culture system of primary hepatocytes and primary Kupffer cells to evaluate the cytotoxicity by the CCK8 cytotoxicity assay and it has been found that 10 μg/mL QTHX-CS was not toxic to cells. So, the 10 μg/mL QTHX-CS concentration was considered the best drug concentration for the experiment. 0, 5, 7.5, 10 μg/mL QTHX-CS drug concentration were set up as NASH group (NASH), low-dose group (low), middle-dose group (middle), and high-dose group (high) respectively.

**Figure 4.**
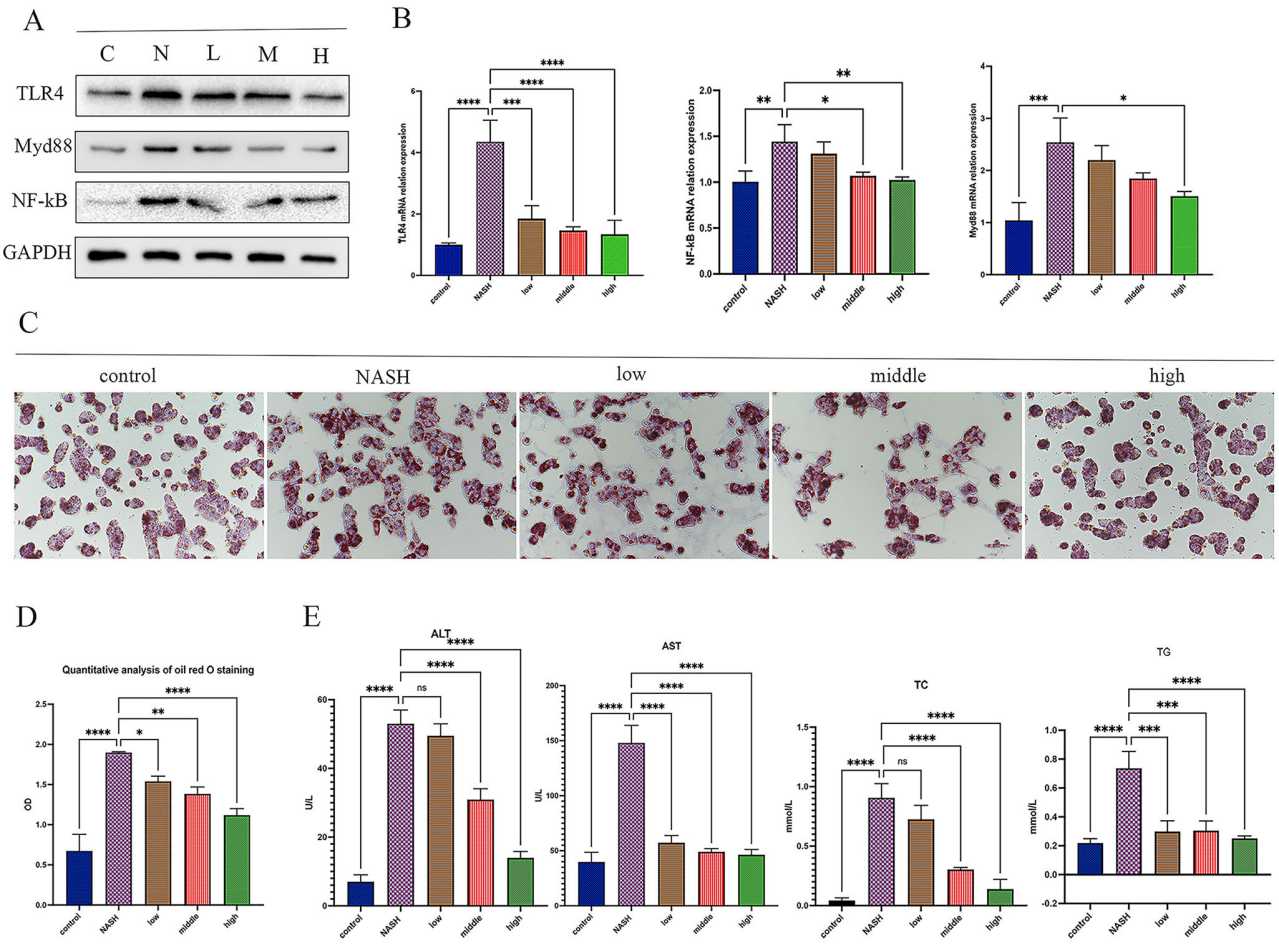
QTHX regulates the SOCS1/TLR4/NF-κB pathway *in vitro*. (A, B) the WB and PCR results of TLR4, Myd88, and NF-κB in primary hepatocytes (C: control group, N: NASH group, L: low group, M: middle group, H: high group). (C, D) Oil red O staining and Oil red O staining quantitative analysis. (E) AST, ALT, TG, and TC in the primary hepatocytes. ****p < 0.0001, ***p < 0.001, **p < 0.01, *p < 0.05.

The results showed that TLR4, NF-κB, and Myd88 proteins were up-regulated in NASH rat primary hepatocytes, and the QTHX down-regulated the TLR4, NF-κB, and Myd88 proteins (Figure 4(A)), and the qPCR results were consistent with the protein results (Figure 4(B)), indicating that QTHX can inhibit the TLR4/NF-κB cell signalling pathway. At the same time, oil red O staining revealed obvious fat droplets in the cytoplasm of primary hepatocytes in the NASH group compared with the normal control group, but QTHX reduced the fat deposition of primary hepatocytes (Figure 4(C,D)). The secretion of ALT, AST, TC, and TG in primary hepatocytes were tested. Compared with NASH group (ALT: 148 ± 9.22 U/L, AST: 53.02 ± 2.30 U/L, TG: 0.74 ± 0.07 mmol/L, TC: 0.91 ± 0.07 mmol/L), the QTHX high dose group showed a significant decrease (ALT: 46.43 ± 2.76 U/L, AST: 13.96 ± 1.08 U/L, TG: 0.25 ± 0.01 mmol/L, TC: 0.14 ± 0.05 mmol/L, *p* < 0.01) (Figure 4(E)). These findings confirmed that the QTHX significantly improved the primary hepatocytes’ damage and lipid droplets got smaller, and TLR4/NF-κB pathway may be involved in the anti-inflammatory response of QTHX *in vitro*.

### Transcriptional regulation of RELA

**Figure 5.**
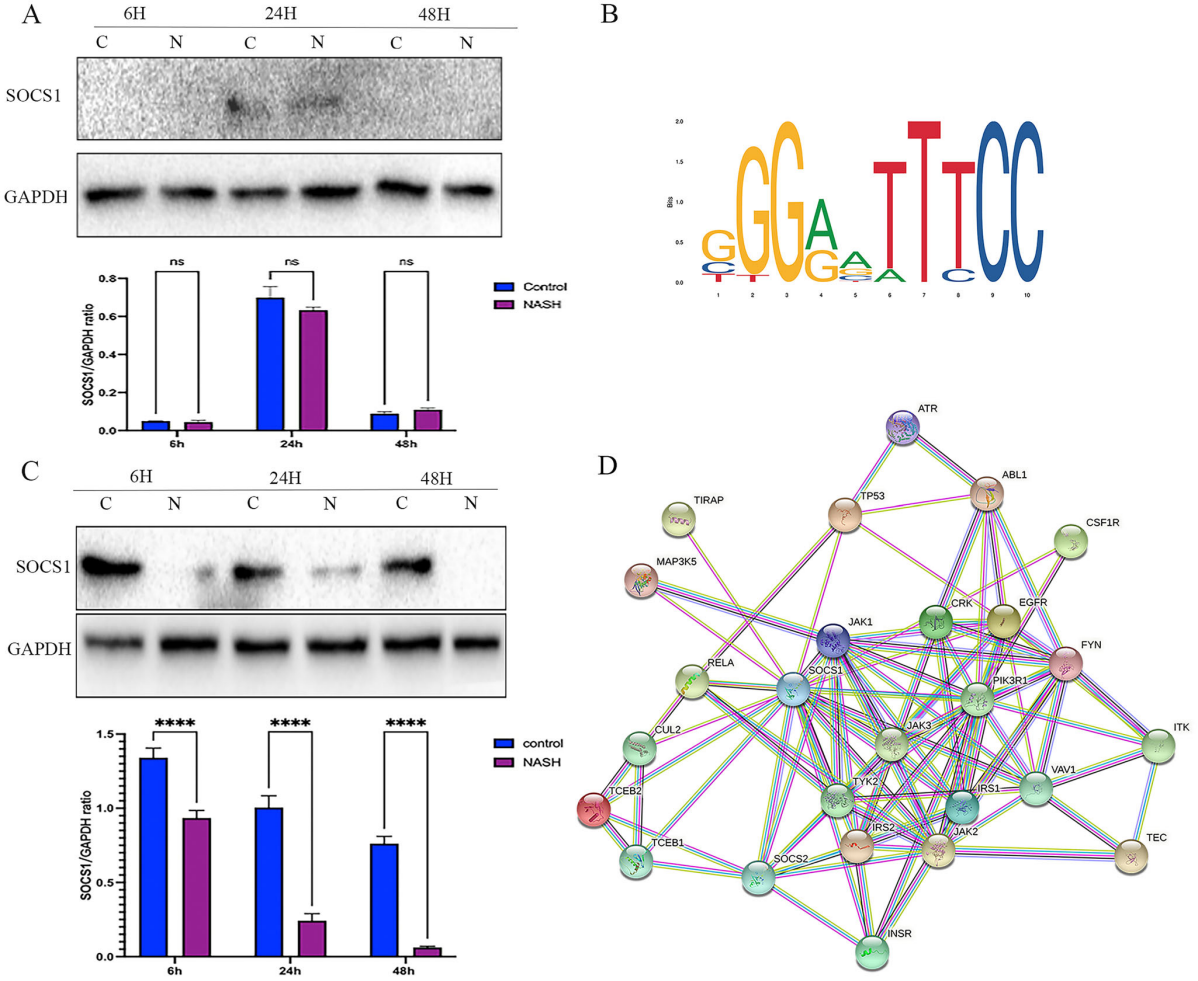
RELA is transcriptionally regulated by SOCS1 (A) The SOCS1 protein in the primary hepatocytes. (B) The recognition motif of RELA from the JASPAR database. (C) The SOCS1 protein in the primary Kupffer cells. (D) PPI network from STING database. **** *p* < 0.0001, *** *p* < 0.001, and ns *p* > 0.05.

To further elucidate the underlying potential mechanism of the role of RELA in the occurrence and development of NASH, the online JASPAR database (https://jaspar.genereg.net/) was used to identify potential target genes. It was founded that the region of the RELA promoter has predicted binding sites for SOCS1. Interestingly, according to the PPI results from the STRING database, SOCS1 was predicted to have a highly reliable interaction with a series of inflammation proteins, such as RELA, JAK1, and MAPK (Figure 5(B,D)). To validate the predicted results and the hypothesized pathway, WB was used, to confirm the expression of SOCS1 in primary hepatocytes and Kupffer cells. Protein changes in SOCS1 in primary hepatocytes and Kupffer cells from NASH and the control group after 6, 24, and 48 h was recorded. It was founded that SOCS1 was not detected in primary hepatocytes after 6 and 48 h, although the level of SOCS1 protein was not statistically significant in the treatment and control in 24 h post-treatment (*p* > 0.05) (Figure 5(A)); The level of SOCS1 protein in NASH primary Kupffer cells was significantly decreased (Figure 5(C)), revealing that KCs play a major role in mediating the SOCS1/TLR4/NF-κB immune-inflammatory pathway.

### TLR4/NF-κB signalling and SOCS1 expression

**Figure 6.**
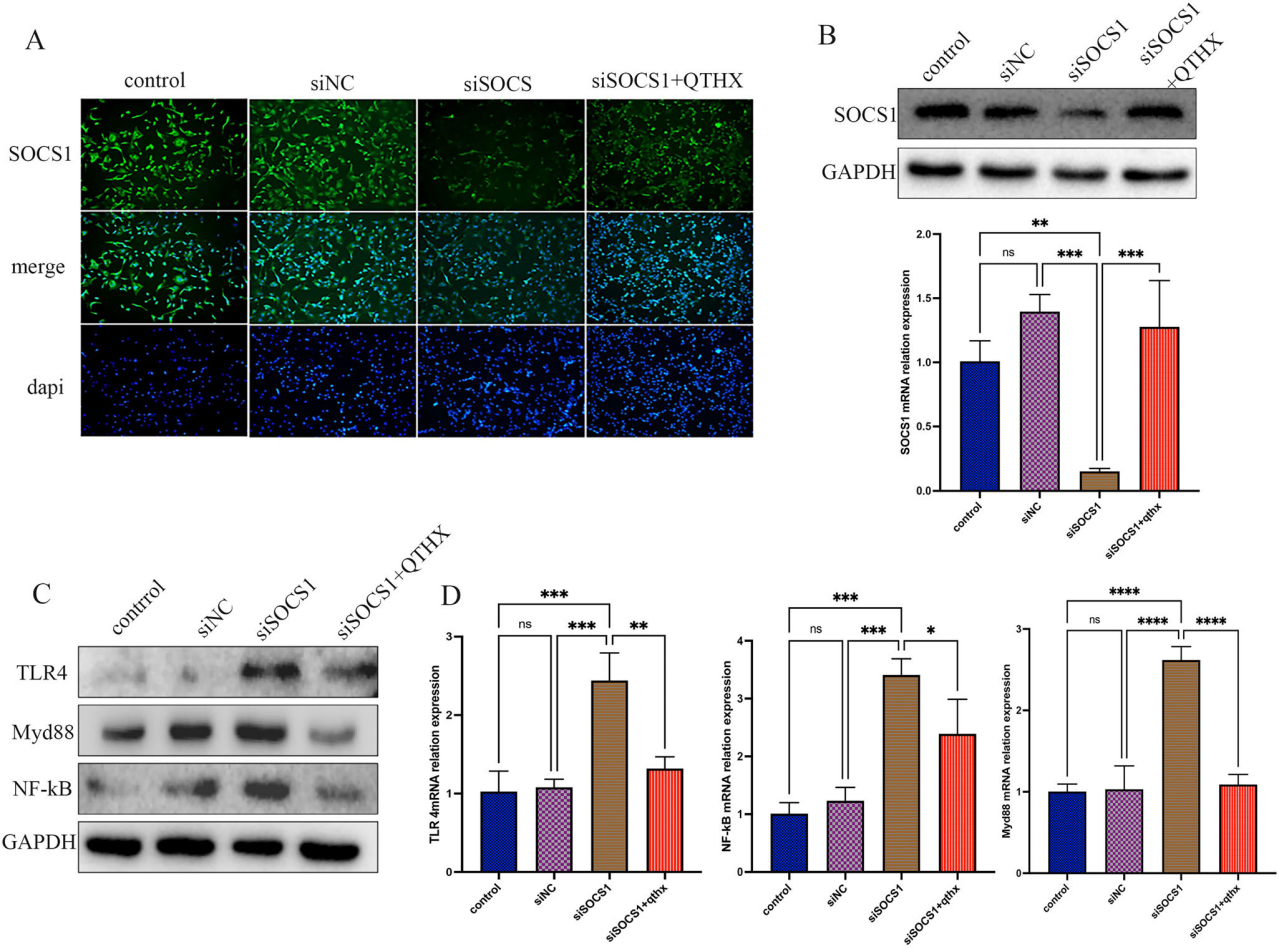
TLR4/NF-κB signalling regulates SOCS1 expression in Kupffer cells. (A, B): the WB, qPCR, and immunofluorescence of SOCS1 siRNA treatment of primary KCs. (C, D) when SOCS1 was silenced, the expression of TLR4, Myd88, NF-κB. ****p < 0.0001, ***p < 0.001, **p < 0.01, *p < 0.05, and ns *p* > 0.05.

It is well known that TLR4/NF-κB signalling-mediated Kupffer cell activation plays an important role in the development of NASH. In this study, the probable influence of TLR4/NF-κB signalling on SOCS1 activation in Kupffer cells was studied. By incubating primary Kupffer cells with SOCS1 siRNA, it was observed that SOCS1 siRNA reduced SOCS1 protein as well as mRNA concentration, as documented by immunofluorescence and qPCR analyses (Figure 6(A,B)). In parallel, the effect of SOCS1 siRNA on Kupffer cell TLR4/NF-κB expression was analysed, and it was found that the expression of TLR4, Myd88, NF-κB was significantly increased (*p* < 0.05) (Figure 6(C,D)), indicating that SOCS1 has a negative feedback regulation effect on TLR4/NF-κB signalling. After the treatment of QTHX (10 μg/mL), the expression of TLR4, Myd88, and NF-κB was significantly reduced (*p* < 0.05), and the analysis showed that QTHX treatment increased the expression of SOCS1 in Kupffer cells.

## Discussion

Traditional Chinese herbal medicine (TCM) has been proven to be effective in many diseases and disorders in clinical practice, but the pharmacological mechanisms remain to be elucidated (Mridha et al. 2017; Zhang CH. et al. 2020; Zhang Y. et al. 2020). The research on the mechanism of Chinese medicine herbal is complicated, and we still don’t have the mature tools to explore the mechanisms of the efficacy of Chinese medicine herbal (Chen et al. 2020). Therefore, by conducting a network pharmacology (NP) analysis, it was found that the potential target might be the key to better to study the synergy between the individual compounds in a TCM to determine the combination of active compounds that is most biologically effective. (Liang et al. 2021). NP is an innovative strategy of picking specific signal nodes through the network analysis of biological systems in a network database to make a targets-drug-diseases molecular network based on the theory of systems biology. It is also an emerging interdisciplinary subject with great advantages in interpreting the pharmacologic mechanisms of TCM with multiple components, targets, and pathways. It is extremely consistent with the theoretical thought of the holistic philosophy of TCM (Nogales et al. 2022). In this study, NP was analysed to find the mechanisms of action of QTHX-treated NASH that mainly involves oxidative stress, membrane raft, membrane microdomain, membrane region, nuclear receptor activity, etc. The mitochondria, the site of cellular energy synthesis, regulate oxidative stress, and plays a key role in cell death (Drake et al. 2003). Therefore, protection by antioxidants against oxidative stress to mitochondria may prove to be beneficial in delaying the onset or progression of diseases (Mancuso et al. 2006). Interestingly, in this network, pharmacology analysis found that the core targets of QTHX in the treatment of NASH include RELA gene, and its function related to lipid metabolism and immune response in liver. This result agrees with the mechanism of our previous study that QTHX can reduce the immune response by regulating the SOCS1/TLR4/NF-κB cell signalling pathway in NASH (Zhang Y. et al. 2020). So, NP gives us a chance to find and verify the key targets for explaining how QTHX regulated the SOCS1/TLR4/NF-κB cell signalling pathway to treat NASH.

The liver is a very complex organ consisting of hepatic parenchymal cells (HPCs) and non-parenchymal cells, including hepatocytes, liver sinusoidal endothelial cells (LSECs), Kupffer cells (KCs), and hepatic stellate cells (HSCs). Increasing evidences suggest that cell-to-cell signal transduction played a vital role in NASH progression and regression (Kazankov et al. 2019; Li et al. 2020). In NASH animal models, the activated KCs with large lipid droplets often gather around hepatocytes and induce the production of inflammatory factors in the case of lipid overload, KCs can make intercellular communication between hepatic cells in liver diseases, it has been demonstrated that KCs also regulate their apoptosis (Yuan et al. 2017). KCs is a macrophage, playing an important regulatory role in liver inflammation, insulin resistance, and fatty liver diseases. Also, research found that the cell structure in NASH patients has undergone significant changes, and KCs are privileged first responder cells in hepatocyte proliferation and liver degeneration (Xiong et al. 2019). The activation of KCs alters hepatic inflammatory cells and chemokine recruitment (Tosello-Trampont et al. 2012), and then activates hepatocytes TLR4/NF-κB inflammation signalling pathways and promotes liver inflammation and fibrosis (Zhang Y. et al. 2020). The inhibition of the inflammatory response of KCs contributes to the treatment of NASH (Mridha et al. 2017). Therefore, in this study, the primary hepatocytes, and KCs were co-cultured to establish a NASH cell model *in vitro*. At present, there are many methods for isolating hepatocytes, such as the liberase-based perfusion technique in combination with low-speed centrifugation and magnetic-activated cell sorting (MACS) was used to isolate and purify HCs, KCs, and LSECs (Liu X et al. 2017). However, many studies chose to use *in situ* perfusion to separate hepatocytes (Mohar et al. 2015; Aparicio-Vergara et al. 2017). In this study, collagenase *in situ* perfusion was used to separate primary hepatocytes and KCs in one step, and the NASH cell model of primary hepatocytes and KCs were successfully established and prepared for exploring mechanism of action.

SOCS1, the most potential member of the SOCS family, can be expressed as a negative regulator that binds to multiple signalling proteins or in a secreted form possessing unique immune suppressive functions (Liau et al. 2018). Research showed that genetic deletion of SOCS1 leads mice to die from inflammation and liver necrosis, in addition, SOCS1 is a potent inhibitor of many cytokines such as TLR ligands, INF-γ, IL-1β, IFNα/β/γ can regulate the inflammation and immune responses (Liu et al. 2008). During the development of NASH, toll-like receptors-4 (TLR4) is one of the major up-regulated genes in NASH. Mechanistically, TLR4 may trigger myeloid differentiation primary response protein 88 (myD88) *via* TIRAP in hepatocytes under inflammatory reaction (Ju et al. 2018; Ciesielska et al. 2021). TLR4 can also activate RELA gene transcription protein NF-κB and induce a strong inflammatory response, adipose accumulation stimulates the overexpression of TLR4 in KCs, activates NF-κB through transcription factors such as MyD88-TIRAP-MAPK, and triggers the pro-inflammatory factors such as MCP-1, TNF-α, and IL-1β. Meanwhile, the triggering of TLR4 also induced SOCS1 protein expression in KCs (Fujimoto and Naka 2010). It’s worth considering how SOCS1 regulates the development of NASH. As reported before, many signal molecules can induce the expression of SOCS1, including IL-2, IL-4, IL-7, IL-10, IL-15, type I and type II IFNs, TNF-α, and colony-stimulating factor (CSF), etc. Meanwhile, SOCS1 inhibits the transmission of cytokine signals (Chandrakar et al. 2020). The main function of SOCS1 is to regulate the interferon signalling pathway, T cell differentiation, and proliferation, inhibit excessive inflammation, regulate the function of dendritic cells, and reduce the production of autoimmune antibodies in the immune response (Ilangumaran et al. 2017). Supporting this concept, SOCS1 expression has been reported to be up-regulated in KCs, which negatively regulate NF-κB and can inhibit activated M1 macrophages (Cheng et al. 2019; Yu et al. 2021). In this study, we verified that the SOCS1 directly related to the progression of hepatic inflammation, and QTHX mainly increases the expression of SOCS1 and decreases the expression of TLR4, NF-κB as well as myd88 in KCs. It’s a key mechanism to explain that how QTHX significantly reduced the fat deposition and inflammation of primary hepatocytes.

The QTHX contains numerous alkaloids and biologically active factors, which interact with each other to regulate inflammation and lipid metabolism in different ways and plays role in the NASH treatment. There is direct evidence, that saikosaponins A and D in *Bupleurum scorzonerifolium* have anti-inflammatory effects by inhibiting the activity of NF-κB (Liu et al. 2017); *Poria cocos* polysaccharides can inhibit the activation of the aortic TLR 4/NF-κB pathway and reduce inflammatory factors and blood lipid levels (Li et al. 2021); *Pinellia ternata* contains a large number of sterols, which can regulate T cell receptor signalling pathway and inflammatory factors (Lyu et al. 2020). *Citrus sinensis* contains flavonoids that inhibit NF-κB and MAPK pathways and inhibit the anti-inflammatory activity of RAW 264.7 cells stimulated by LPS (Son et al. 2020). *Scutellaria baicalensis* can regulate lipid metabolism and liver function (Yang et al. 2019). It’s a challenge to explore and verify the specific mechanism of action in diseases when the prescription including different individual traditional Chinese medicine herbals, due to that the target of the individual traditional Chinese medicine herbal was heterogeneous. Herein, a key target of QTHX reducing inflammatory response in NASH was demonstrated by the NP and experiment *in vitro*. However, the liver is a sophisticated and complex organ, and the related targets in the occurrence and development of NASH are complex and diverse (Miyao et al. 2015; Wang et al. 2021). So, it is still a challenge to show the overall view of the mechanism of TCM prescription in diseases therapy.

## Conclusions

QTHX can significantly inhibit fat accumulation in primary hepatocytes and inflammatory response in KCs by mediating SOCS1/TLR4/myd88-NF-ĸB cellular signalling pathway. Our findings provided a novel viewpoint on QTHX's NASH-treatment mechanism, as well as a reference for future research on the efficacy, mechanism, and potential clinical applications of QTHX.
